# Anterior debridement, bone grafting and fixation for cervical spine tuberculosis: an iliac bone graft versus a structural manubrium graft

**DOI:** 10.1186/s12891-022-05177-0

**Published:** 2022-03-11

**Authors:** Shuang Xu, Gaoju Wang, Jin Yang, Shuai Zhang, Yueming Song, Qing Wang

**Affiliations:** 1 Department of Orthopedics, Orthopedic Research Institute, West China Hospital, Sichuan University, Chengdu, 646000 Sichuan China; 2 Department of Orthopedic Surgery, Affiliated Hospital of Southwest Medical University, No. 25 of Taiping Road, Luzhou, 646000 Sichuan China

**Keywords:** Manubrium, Autograft, Cervical tuberculosis, Kyphosis, Instrumentation

## Abstract

**Background:**

Anterior debridement, decompression, bone grafting, and instrumentation are safe and effective techniques for patients with lower cervical spine tuberculosis. However, there is no consensus regarding the methods for using autogenous bone grafts. The purpose of this retrospective study was to compare the clinical outcomes of anterior surgical management for cervical spine tuberculosis by using an iliac bone graft versus a structural manubrium graft.

**Methods:**

From January 2009 to September 2018, 23 patients with cervical spine tuberculosis were treated with anterior debridement, autogenous structural bone grafting and fixation at our spinal department. The patients were divided into 2 groups according to the different graft materials, namely, iliac crest bone grafts (Group A) and structural manubrium grafts (Group B). The clinical and radiographic results of the 2 groups were analyzed and compared.

**Results:**

The mean duration of follow-up was 24 months. Bony fusion was achieved in all patients without failure of internal fixation. There were no significant differences between the two groups with respect to the operation time, blood loss, fusion time, neurological outcomes, or postoperative local Cobb angle (*P* > .05). However, the donor site complication rate in Group A was greater than that in Group B. The postoperative ambulation time in Group A was later than that in Group B. The mean visual analog scale (VAS) score for donor site pain in Group A was higher than that in Group B at 1 week after surgery (*P* < 0.05). However, there was no significant difference between the 2 groups at the last visit (*P* > .05).

**Conclusion:**

Both iliac bone grafts and sternal manubrium grafts can effectively reconstruct anterior column defects in anterior surgery. However, structural sternal manubrium autografts cause fewer complications associated with donor site morbidities than iliac bone grafts.

## Introduction

Although anti-tuberculosis chemotherapy and improved nutrition have resulted in excellent functional outcomes in some patients, patients with severe kyphosis, those developing neurological deficits, or those with therapeutically refractory disease need surgical intervention [1, 2]. It is generally accepted that the aim of surgical treatment of spinal tuberculosis is debridement of the lesion, amelioration of spinal cord compression, and correction of spinal deformity. Anterior surgery is a commonly performed procedure for treating lower cervical spine tuberculosis [3]. However, the reconstruction of bony defects after debridement remains a major challenge.

An autologous iliac bone graft is regarded as the gold standard surgical strategy due to its superior osteoinductive and osteoconductive properties. However, the high incidence of associated donor site morbidities remains a major concern [4]. To prevent these complications, multiple alternative bone grafts, such as a vascularized fibular graft, allograft, titanium mesh and synthetic interbody cage, have been developed as substitutes for autologous iliac bone. The advantages and disadvantages of these surgical options have been described extensively in the literature [5–8].

In recent years, the manubrium has been considered a novel source of cancellous autografts for cervical fusion. Several studies have reported that cancellous bone derived from the manubrium is associated with satisfactory fusion and fewer donor site complications [9, 10]. None of the reports have described harvesting of a manubrium autograft that can serve as a structural graft. In this study, we describe a method using structural bone obtained from the manubrium for treating cervical spine tuberculosis. With iliac bone grafting as the control treatment, we compared the clinical and radiographic outcomes associated with the two treatments for lower cervical spine tuberculosis.

This retrospective study was performed to explore the feasibility and clinical efficacy of anterior surgical management for cervical spine tuberculosis by using a structural manubrium graft.

## Materials and methods

### Population data

From January 2009 to September 2018, 23 patients with cervical spine tuberculosis who underwent surgery in our hospital were initially reviewed retrospectively. The diagnosis of tuberculosis was first made on the basis of medical history, clinical examination, laboratory results (erythrocyte sedimentation rate, ESR, C-reactive protein, CRP), radiologic imaging (X-ray, CT, and MRI), and drug response. A definitive diagnosis was made by histological examination and/or by polymerase chain reaction (PCR) of the resected tissue. The indications for surgery were severe kyphosis of the cervical spine, spinal instability, and neurologic deterioration. The inclusion criteria were as follows: (1) patients with lesions involving the lower cervical vertebrae (C3-C7), (2) patients who underwent debridement, autogenous bone grafting and instrumentation by an anterior-only approach, (3) patients with a definitive diagnosis of tuberculosis by pathological examination or PCR, and (4) patients with a minimum 2-year follow-up. The exclusion criteria were as follows: (1) damage to multiple cervical vertebrae; (2) tuberculosis lesions in the posterior column; and (3) loss to follow-up due to any reason. According to the different bone grafting methods, the patients were divided into the following two groups: the iliac bone graft group (Group A) and the structural manubrium graft group (Group B).

### Preoperative examination and preparation

All the patients underwent anti-tuberculosis treatment by daily oral administration of isoniazid 300 mg, rifampin 450 mg, ethambutol 750 mg, and pyrazinamide 750 mg for at least 2–4 weeks preoperatively based on a previous study [11]. The malnourished patients were given nutritional support therapy to improve their preoperative condition.

**Fig. 1:**
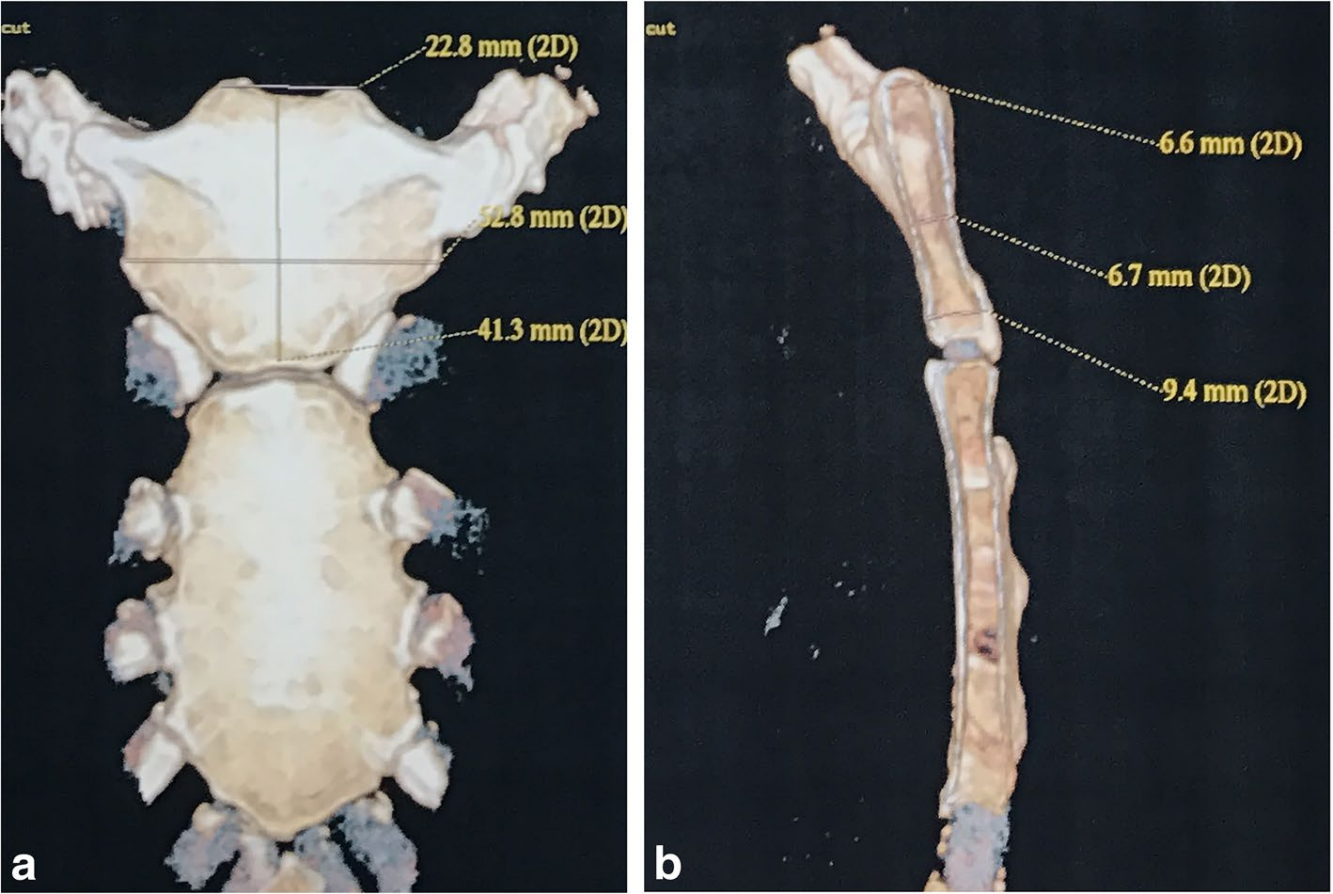
In the workstation, the breadth, height and thickness of the manubrium were measured (**a**, **b**)

Before surgery, the patients were informed about the various options for anterior cervical reconstruction: either (1) an iliac bone graft, (2) a structural manubrium graft, or (3) titanium mesh cages with an allograft. The advantages and disadvantages of each of these options were discussed extensively by the surgeon with the patients, and the patients made the decision and signed a consent form. Three-dimensional computed tomography (3D-CT) was performed to investigate the anatomy of the manubrium in all the patients who selected a sternal graft. In the workstation, the breadth, height and thickness of the manubrium were measured as described by Peng [12] (Fig.1). In the case of the presence of an extremely small or malformed manubrium, a substitutable procedure was considered to avoid pitfalls.

### Surgical technique

All the operations were performed by one of the three senior surgeons at our institute. The surgery was divided into 3 steps. The first step was radical debridement of cervical spine tuberculosis lesions through the Smith-Robinson anterolateral approach. The height of the anterior gap was measured after washing the wound repeatedly. Step 2 of the surgery was harvesting a bone graft. The iliac bone harvesting technique was performed in Group A according to the method described in previous literature [4]. Structural bone was harvested from the manubrium in Group B as follows. A longitudinal or transverse 3-cm incision was made directly over the manubrium. Dissection was performed through the subcutaneous tissue to the periosteum. The anterior aspect of the manubrium was exposed to the medial limits of the sternoclavicular joints. A sternal block was harvested using piezosurgery, which was limited by the sternoclavicular joints laterally and 0.5 cm above the sternal angle cephalic end and below the suprasternal notch. Then, hemostasis was performed carefully by bipolar electrocoagulation. The defect of the manubrium was reconstructed with a gelatin sponge and the remaining morselized bone. Finally, a drainage tube was placed, and the incision was closed in layers.

**Fig. 2:**
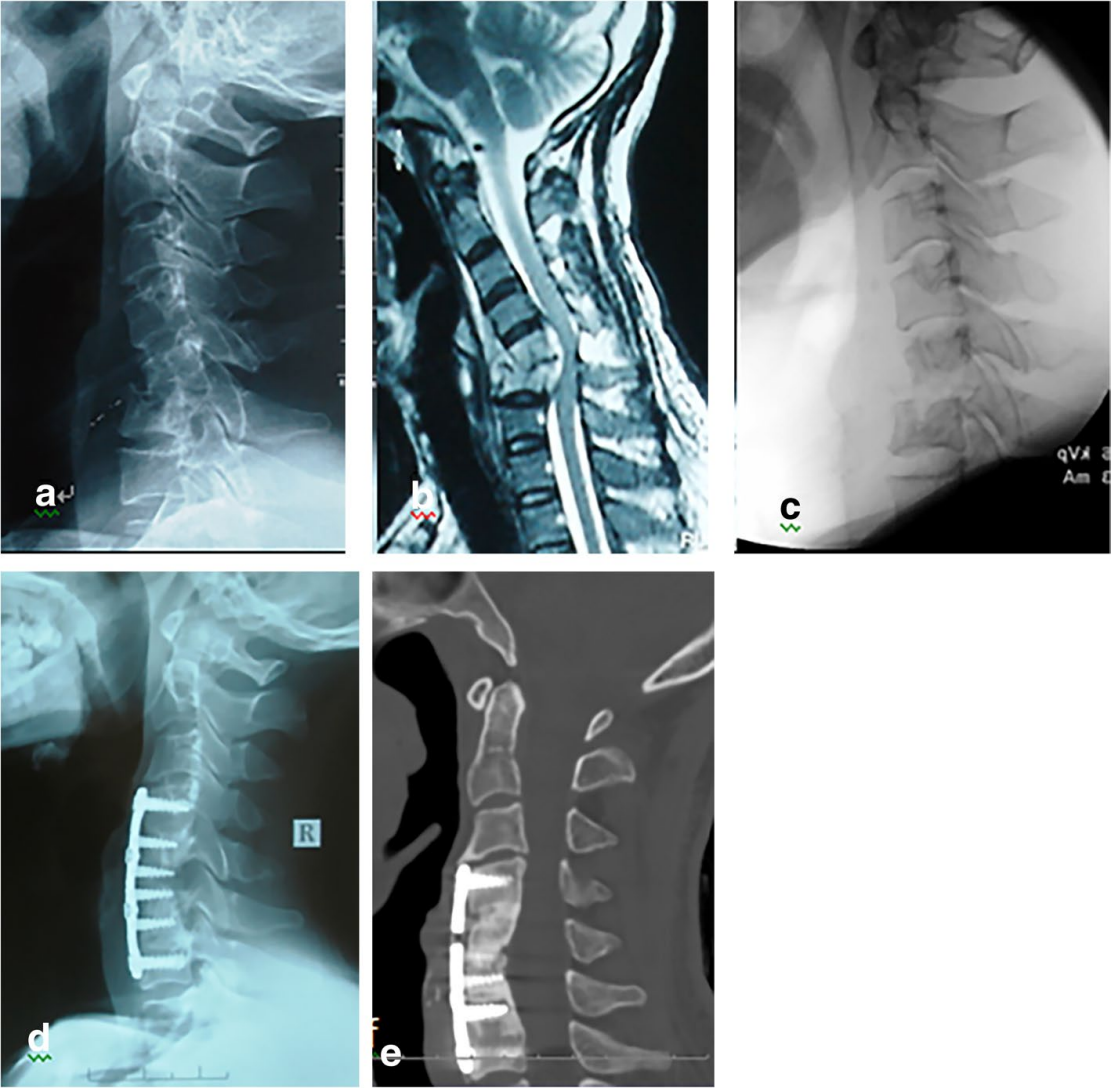
A 46-year-old male patient with cervical spine tuberculosis (C5–6). **a**-**c** Preoperative radiography and MRI showed destruction of C5, C6 vertebrae with kyphosis and spinal cord compression. **c** X-ray showed reduction of cervical kyphosis after intraoperative traction. **d** X-ray demonstrated anterior debridement, iliac bone grafting and internal fixation from C4 to C7. **e** CT scan at 12 months of follow-up showed bone fusion at the interface

**Fig. 3:**
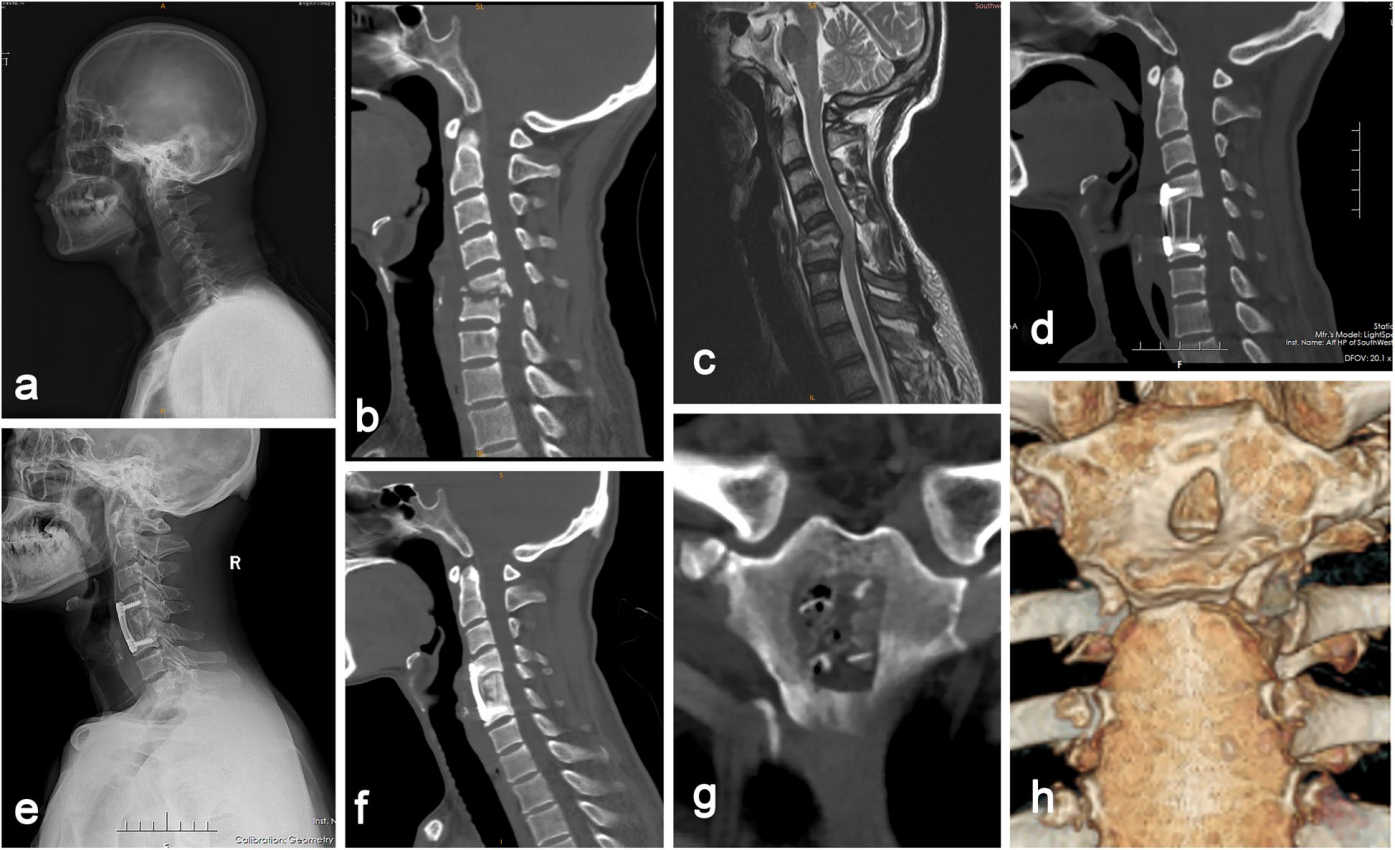
A 46-year-old male patient with cervical spine tuberculosis (C5–6). **a**-**c** Preoperative radiography, CT and MRI showed significant cervical kyphosis, C5 and C6 vertebral body destruction, epidural abscess formation and spinal cord compression. **d** Postoperative CT showed that cervical kyphosis improved after anterior debridement, structural manubrium grafting and anterior plate fixation. **e** Radiography at 3 months of follow-up showed bony fusion at the graft-host junction. **f** CT scan at 12 months of follow-up showed that correction of kyphosis was maintained and that the implants were in good position. **g**-**h** Postoperative 3D-CT showed the scope of the defect of the manubrium, which decreased 1 year after the operation

The final step included grafting and fixation. An iliac bone autograft was used to reconstruct the anterior defect in Group A (Fig. 2), while a structural manubrium autograft was placed in the gap to reconstruct the anterior defect in Group B (Fig. 3). Then, a locking plate-screw system of appropriate length was used to achieve anterior cervical fixation. After hemostasis and washing were performed, a deep drainage tube was placed, and the incision was closed in layers.

### Postoperative care

The drainage tube was removed when the volume of drainage was less than 50 ml/24 h. The patients were allowed to ambulate with the support of a neck brace after 3 days of surgery, which was used for 12 weeks. Anti-tuberculosis chemotherapy, which was the same as the preoperative regimen, was continued for 3 months postoperatively, followed by a regimen of isoniazid, rifampicin, and ethambutol for another 12–15 months [3].

### Clinical evaluation and follow-up

Patient follow-up was performed at 3 months, 6 months, and 1 year postoperatively, and all the findings were recorded by the surgeon. The primary outcomes measured were the ESR, Cobb angle, VAS score, complications, and fusion rate. At each follow-up, lateral X-ray films or CT was performed. Fusion was assessed by the presence of bridging trabecular bone and the absence of radiolucency at the junction between the graft/cage and opposing vertebra [13].

### Statistical analysis

Data analysis was performed using SPSS for Windows version 19.0 (IBM, New York, New York, USA). Paired t tests and independent-samples t tests were employed for intragroup and between-group comparisons of the continuous variables between the 2 groups, respectively. Categorical data were analyzed with the chi-square test. Values of *P* < 0.05 were considered to indicate significant differences.

## Results

According to the inclusion criteria, a total of 23 patients were enrolled in this study. There were 15 patients (10 males and 5 females) with a mean age of 38.5 years in Group A, and all the patients underwent anterior debridement, iliac bone grafting and fixation. In Group B, there were 8 patients (4 males and 4 females) with a mean age of 49.5 years, and all of them were managed with anterior debridement, structural sternal block grafting and instrumentation. All the patients were followed up for an average of 24 months. The basic information of the patients in the two groups is shown in Table 1.

**Table 1:** The basic information of patients in two groups

Characteristic	Group A(15 patients)	Group B(8 patients)	*p*
Age	47.6.5 ± 8.4	50.7 ± 13.4	0.532
Sexal(m/w) pathogenic level	10/5	6/2	0.888
C3–4	1	0	
C4–5	4	2	
C5–6	7	5	
C6–7	3	1	

The clinical data of the patients in the two groups are shown in Table 2. The operation time and blood loss between the two groups were not significantly different (*P* > 0.05). In both groups, the ESR gradually decreased after the operation, and it was restored to the normal level at 3 months postoperatively. There was no significant difference between the two groups (*P* > 0.05). X-ray and/or CT examination showed that bone graft fusion was achieved in all cases. The mean fusion time showed no significant difference between the two groups (*P* > 0.05). Compared with the preoperative local Cobb angles, the postoperative values were increased in both groups and slightly decreased at the final follow-up. However, there was no significant difference between the two groups (*P* > 0.05). The neurological outcomes of Groups A and B are presented in Table 3. The American Spinal Injury Association (ASIA) grade B/C/D in the patients in the 2 groups was improved 1 to 2 grades after surgery. No statistically significant difference was found between the 2 groups postoperatively. The mean visual analog scale (VAS) score for donor site pain in Group A was greater than that in Group B at 1 week after surgery (*P* < 0.05). At the final follow-up, there was no significant difference in the VAS score between the two groups. The mean ambulation time in Group A was greater than that in Group B after surgery (*P* < 0.05).

**Table 2:** The clinical data of patients in two groups

Characteristic	Group A(15 patients)	Group B(8 patients)	*p*
**Surgical time (min)**	173.3 ± 20.8	179.8 ± 46.4	0.631
**Blood Loss (ml)**	323.4 ± 105.1	281.0 ± 84.2	0.336
**Ambulation time (day)**	6.7. ±1.4	3.8 ± 0.8	0.000
**Duration of follow-up(m)**	24.0 ± 5.7	25.8 ± 7.6	0.509
**Fusion time(m)**	4.3 ± 1.6	4.4 ± 1.4	0.917
**ESR**			
Preoperative	55.2 ± 19.1	61.4 ± 19.5	0.170
Postoperative	21.8 ± 9.9^a^	26.8 ± 6.2^a^	0.144
Final follow-up	11.4 ± 6.3^a^	13.5 ± 4.3^a^	0.371
**Cobb(°)**			
Preoperative	18.1 ± 8.2	16.3 ± 8.2	0.577
Postoperative	−2.4 ± 2.6^a^	− 2.1 ± 2.8^a^	0.746
Final follow-up	−1.0 ± 2.2^a^	− 1.4 ± 2.5^a^	0.732
**VAS (donor-site)**			
A week Post-op	3.4 ± 0.9	2.3 ± 0.9	
3-months Post-op	1.6 ± 0.7^b^	1.1 ± 0.7^b^	0.005
Final follow-up	0.7 ± 0.5^b^	0.6 ± 0.5^b^	0.070
Complication**(donor-site)**	3	0	0.569

VAS 10-point visual analog scale

^a^ Compared with preoperative (*P* < 0.05)

^b^Compared with A week Post-op (*P* < 0.05)

**Table 3:** The neurological outcomes of patients in two groups

ASIA	Group A					Group B				
	A	B	C	D	E	A	B	C	D	E
Preoperative		1	2	8	4			3	5	0
Postoperative				2	13				1	7

In Group A, three mild complications occurred. One patient developed superficial wound infection at the donor site. It was treated successfully with oral antibiotics and dressing changes. Two patients experienced chronic donor site pain, which was treated with nonsteroidal anti-inflammatory drugs, and it was markedly improved 3 months after surgery. In Group B, none of the patients complained of donor site pain, and narcotic pain medication was not required for donor site pain after discharge.

## Discussion

In clinical practice, patients with cervical spine tuberculosis often develop severe bone destruction, spinal cord compression, and/or kyphotic deformity. Conservative treatment alone often fails to relieve spinal cord compression, improve nerve dysfunction, and prevent spinal deformity. Patients with this disease should be treated with surgical decompression, bone grafting, and fixation. Anterior surgery has become the standard procedure for treating cervical spine tuberculosis because cervical spine tuberculosis mainly invades the spinal anterior column [3]. The procedure offers the advantages of removing the focal point of the disease directly and spinal cord and nerve decompression, with minor surgical invasiveness and effective kyphosis correction. However, the large intervertebral gap created by debridement causes instability of the cervical spine. Therefore, reconstruction of the anterior spinal column is necessary [14].

Autologous tricortical bone grafts are widely used as grafting materials for reconstructing the spine, and they are considered the gold standard material because they provide an osteoconductive, osteoinductive, and osteogenic substrate for filling bone voids. Hassan et al. [15] reported the outcomes of 16 patients with cervical spinal tuberculosis who underwent anterior debridement, autogenous iliac bone grafting, and instrumentation. Bony fusion and marked improvement in kyphosis were achieved in all patients at the latest follow-up. However, donor site complications, such as pain at the donor site, nerve injury, hematoma, infection, and fracture, have been reported in up to 40% of cases [4]. The most common complication associated with the harvesting of an iliac bone is pain at the donor site. In the study by Wael Koptan et al. [16], the anterior column was reconstructed with an autogenous iliac bone graft in 14 patients with cervical spine tuberculosis; four patients had persistent pain at the donor site, and one patient had a large hematoma (35%). In our cohort, one patient developed superficial wound infections at the donor site, and 2 patients developed chronic donor site pain after surgery (20%).

To retain the advantages of an autogenous graft and minimize donor site morbidities, a sternal graft has been applied in anterior cervical fusion (ACF) procedures [9–12]. The cancellous bone harvested from the sternal manubrium with an interbody cage has been used for ACF, and the results showed that the sternal manubrium provides a viable alternative to iliac crest grafting and provides the advantages of autograft fusion without the complications associated with iliac crest graft harvesting [9, 10].

In the present study, a structural sternal autograft was harvested for anterior reconstruction of the cervical spine. In our experience to date, the structural sternal autograft has the following advantages. First, it has a high osteogenic potential. In our group of 8 patients, all the patients achieved satisfactory bone fusion. Second, both ACF and bone harvesting were performed in a single operative field, which can save the time and labor needed to prepare separate sterile fields and reduce the chance of intraoperative pollution. Third, there was a low complication rate at the donor site. In the present study, there was no hematoma formation, infection, injury to the great vessels or pleura or chronic pain in Group B, while 3 patients (20%) had donor site complications in Group A. Significant pain at the iliac donor site has been shown to affect early ambulation. Patients who stay in bed for a longer time may experience complications such as pneumonia and deep vein thrombosis. In contrast, there was no postoperative pain in the chest area in the patients with bone harvested from the manubrium, which allowed ambulation with a cervical collar 3 days after the surgery.

We also identified some disadvantages of using a structural manubrium autograft. We obtained a two-sided cortical bone compared with a tricortical iliac crest, which may have an insufficient supporting force and lead to fracture. However, no fractures were found in our 8 patients, which may be because we excluded elderly patients with osteoporosis. Another disadvantage was that the amount of bone mass obtained was only adequate for reconstruction of bone defects within two segments, while the iliac crest provides a larger bone mass. In addition, the visibility of the scar may affect the cosmetic result while wearing low-cut clothing. Therefore, it is advisable to avoid this method in young women or in patients who are particularly concerned about their appearance. Finally, there are some potential risks when obtaining bone from the manubrium, such as injury to the great vessels and pleura. Thus, it is necessary to take advantage of piezosurgery to obtain bone because piezosurgery is a recent innovative technique that ensures a selective cut of mineralized tissue while sparing soft tissues.

There are several limitations to this study. The sample size was small and may not adequately explain the advantages of harvesting structural bone from the manubrium. In addition, because the follow-up time was short, the long-term effect of a manubrium bone graft could not be determined; thus, long-term follow-up is needed.

## Conclusion

Anterior debridement, fusion and internal fixation for the treatment of lower cervical spine tuberculosis is an effective and feasible method. Both iliac bone grafts and sternal manubrium grafts can be used to effectively reconstruct the anterior column defects created by completely clearing the tuberculosis focus in anterior surgery. However, structural sternal manubrium autografts may cause fewer complications associated with donor site morbidities than iliac bone grafts. Further studies with a large number of patients and longer follow-up periods are needed.

## Data Availability

The datasets supporting the conclusions of this article are included within the article. The raw data can be requested from the corresponding author on reasonable request.
